# Accuracy of the WHO praziquantel dose pole for large-scale community treatment of urogenital schistosomiasis in northern Mozambique: Is it time for an update?

**DOI:** 10.1371/journal.pntd.0006957

**Published:** 2018-11-15

**Authors:** Pedro H. Gazzinelli-Guimaraes, Neerav Dhanani, Charles H. King, Carl H. Campbell, Herminio O. Aurelio, Josefo Ferro, Rassul Nala, Alan Fenwick, Anna E. Phillips

**Affiliations:** 1 Schistosomiasis Control Initiative, Department of Infectious Disease Epidemiology, Imperial College, London, United Kingdom; 2 Center for Global Health and Diseases, Case Western Reserve University, Cleveland, Ohio, United States of America; 3 Schistosomiasis Consortium for Operational Research and Evaluation, University of Georgia, Athens, Georgia, United States of America; 4 Faculty of Health Sciences, Universidade Católica de Moçambique (UCM), Beira, Moçambique; 5 Ministério da Saúde, Maputo, Moçambique; 6 London Centre for Neglected Tropical Disease Research, Department of Infectious Disease Epidemiology, Imperial College, London, United Kingdom; University of Florida, UNITED STATES

## Abstract

**Background:**

A pioneering strategy developed by the World Health Organization (WHO) for the control of schistosomiasis was the concept of a height-based dose pole to determine praziquantel (PZQ) dosing in large-scale treatment campaigns. However, some recent studies have shown variable accuracy for the dose pole in terms of predicting correct mg/Kg dosing, particularly for treatment of adults. According to the WHO, 91 million adults in 52 countries are targeted to be treated by 2020.

**Methods/Principal findings:**

The present study aimed to test the accuracy of the dose pole in determining PZQ dosage by comparing the number of tablets determined by the dose pole with the number of tablets determined according to total body weight. The analysis included height-for-weight data from 9,827 school-aged children (SAC) and adults from 42 villages in the province of Cabo Delgado in Mozambique. The results revealed that of the 7,596 SAC, 91.8% has received an appropriate dose (30-60mg/Kg), 6% received an insufficient dose (<30mg/Kg) and 2% an excessive dose (> 60mg/Kg). On the other hand, 13.7% out of 2,231 adults were treated inaccurately with 13.5% receiving an insufficient dose and 0.2% an excessive dose. When the percentage of insufficient dosing was disaggregated by gender, the frequency of adult females who were underdosed reached 18.3% versus 10.8% of adult males. Of note, Adult females aged 21–55 years were found to have an underdose frequency of 21.3%, compared to 11.8% of adult males in the same age range. The performance of a proposed modified dose pole was compared using the same dataset of adult Mozambicans. The results showed that the modified dose pole reduced the underdose frequency among adults from 13.5% to 10.4%, and subsequently increased the percentage of optimal dosing from 33.7% to 45.3%.

**Conclusions:**

Our findings highlight the need to update the WHO-dose pole to avoid administration of insufficient PZQ doses to adults and therefore minimize the potential emergence of PZQ-resistant strains.

**Trial registration:**

International Standard Randomized Controlled Trial registry under ISRTC number 14117624

## Author summary

Schistosomiasis control is currently based on large-scale, preventive chemotherapy treatment with praziquantel (PZQ), and this has resulted in a significant reduction in its prevalence and associated morbidity worldwide. Recommended PZQ dosing is 40–60 mg/Kg body weight, but to facilitate large-scale drug administration programs, a dose pole to estimate PZQ doses based on height has been developed by WHO, as height has been shown to accurately correlate with body weight among children in endemic areas. WHO estimates that 206 million people, including 91 million adults, will need to be treated annually across 52 countries by 2020. Published literature indicates that the dose pole has a satisfactory accuracy for children, however it seems to deliver an insufficient dose (<30mg/Kg body weight) to approximately 20% of adults and overweight children. This paper evaluated the accuracy of the PZQ dose pole for the large-scale treatment of urogenital schistosomiasis in Mozambique and discusses the necessity for an update of the current WHO-dose pole based on modifications recommended in recent literature. Preventing underdosing among adults and overweight children should improve treatment outcomes and minimize the potential emergence of PZQ-resistant strains.

## Introduction

Human schistosomiasis is an acute and chronic neglected tropical disease (NTD) caused by six species of parasitic blood flukes of genus *Schistosoma*: *S*. *guineensis*, *S*. *haematobium*, *S*. *intercalatum*, *S*. *japonicum*, *S*. *mansoni* and *S*. *mekongi*. Together, they affect about 250 million people worldwide, with more than 779 million people are at risk of infection [1, 2]. Despite being endemic in 52 countries around the world, approximately 91% of schistosomiasis prevalence is concentrated in Africa, causing severe morbidity, mainly in school-aged children (SAC)[3].

For almost 20 years, schistosomiasis control has been based on large-scale, repeated, preventive chemotherapy treatment with praziquantel (PZQ), with an optimal dose of 40-60mg/Kg bodyweight. The drug is recommended due its safety, acceptable cure rate in low intensity infection, and its currently low cost [4]. However, Taylor *et al*. [5] and King, *et al*. [6] demonstrated that PZQ may have similar effects in slightly lower doses, therefore, a single dose between 30-60mg/Kg has been considered acceptable for schistosomiasis preventive chemotherapy. On the other hand, others have shown variation in the schistosome sensitivity to PZQ in different endemic regions [7, 8]. The current World Health Organization (WHO) guideline for schistosomiasis preventive chemotherapy recommends annual treatment of all SAC as well as at-risk adults in areas with >50% prevalence, treatment of SAC every two years (school-based treatment) as well as high-risk adults in areas with 10–50% prevalence, and treatment of SAC twice during primary school years in areas with 1–10% prevalence [9].

A part of the mass drug administration (MDA) strategy recommended by WHO is the use of the height-based dose pole to estimate appropriate PZQ doses. This low-technology approach is field-applicable and suitable for rapid, large-scale implementation of treatment [10]. In the early 2000s, when the WHO dose pole for schistosomiasis was developed, several studies demonstrated that it could determine an appropriate dose between 30-60mg/Kg in more than 98% of SAC [10–12]. However, recent studies have found a questionable accuracy for the dose pole in determining correct dosage for adults. In South Africa, for example, 34.6% of adult females (16–23 years old) received an insufficient dose of PZQ based on dose pole recommendations [13]. Another study, which evaluated 5,614 rural adult Zimbabweans (15–49 years old), showed a 15% risk of inadequate treatment (< 30mg/Kg) for adults [14]. Interestingly, a common aspect between these two studies was an elevated prevalence of obesity among adults; 40.9% in South Africa and 19.4% in Zimbabwe.

Between 2011 and 2015, a Schistosomiasis Consortium for Operational Research and Evaluation (SCORE) treatment project [15] was carried out in Mozambique [16, 17]. This longitudinal study compared the impact of different treatment strategies in study areas having prevalence of ≥ 21% for urogenital schistosomiasis (*S*. *haematobium*) among SAC. The primary goal of SCORE project was to gain and sustain low levels of infection through preventive chemotherapy. In different study arms, treatment was given in schools or in communities or both to evaluate the impact of alternative treatment approaches [17]. In the final and the fifth year of this study, data were additionally collected in some villages to evaluate the accuracy of the WHO-dose pole for both SAC and adults.

## Methods

### Ethics statement

This study was performed as part of the SCORE project in Mozambique, which was registered with the International Standard Randomized Controlled Trial registry under ISRTC number 14117624 for Mozambique. Informed written consent was obtained from all individuals ≥18 years of age and from parents or legal guardians of children less than 18 years of age. The purpose of the study was explained to all school children and verbal assent was obtained from the children. Permission was also obtained from school headmasters. Ethical clearance was obtained from the National Bioethical Committee for Health of Mozambique (NBCHM) [Comitê Nacional de Bioética para a Saúde (CNBS)], and the survey was conducted according to CNBS guidelines (reference no. IRB00002657). The study protocol was also approved by Imperial College London (ICREC_10_2_2).

### Study population, data management, and analysis

**Fig 1:**
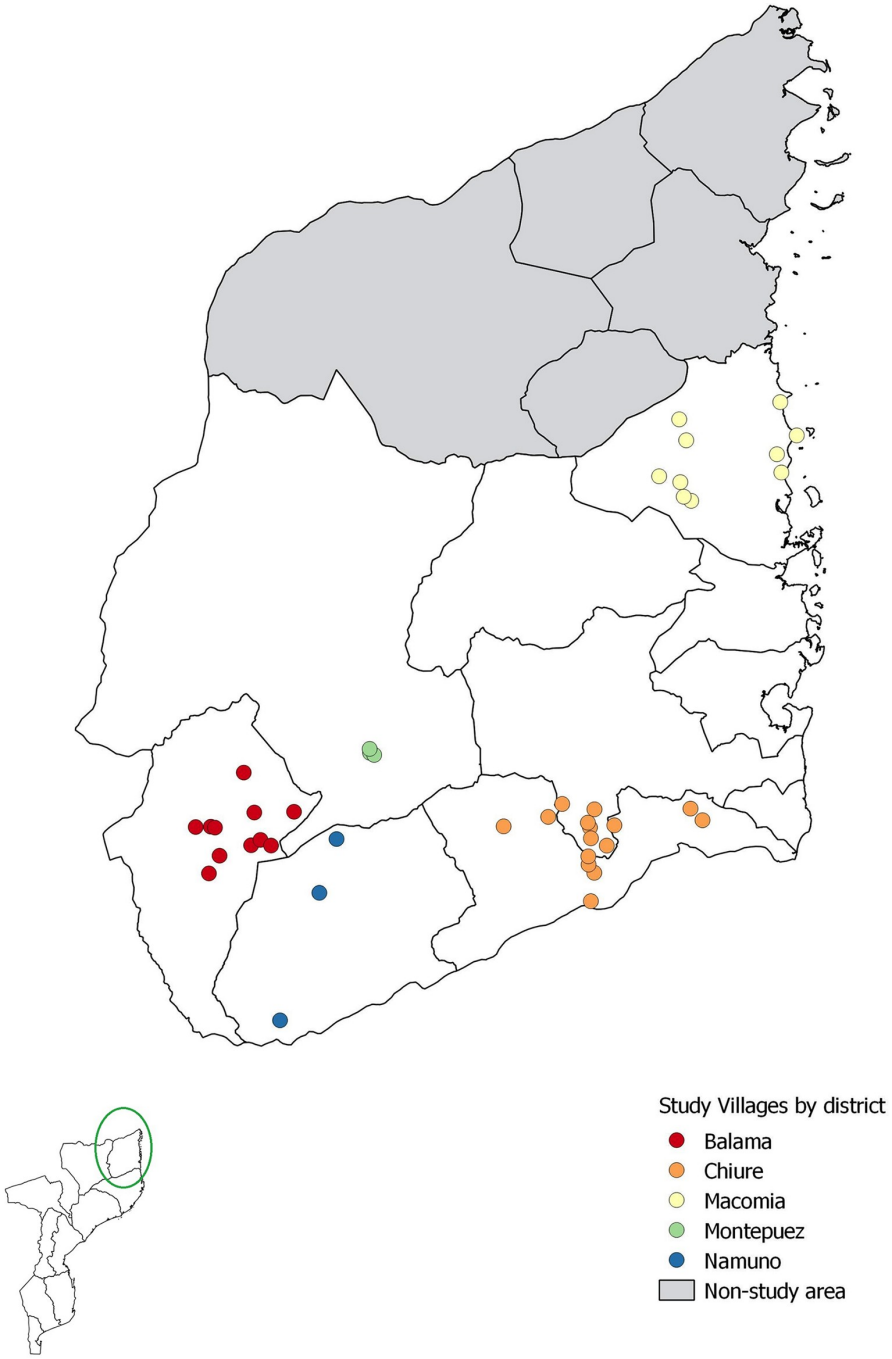
42 villages from 5 districts surveyed in the province of Cabo Delgado, northern Mozambique. We created the map using QGIS and publicly available shapefiles from http://www.diva-gis.org/gdata.

**Fig 2:**
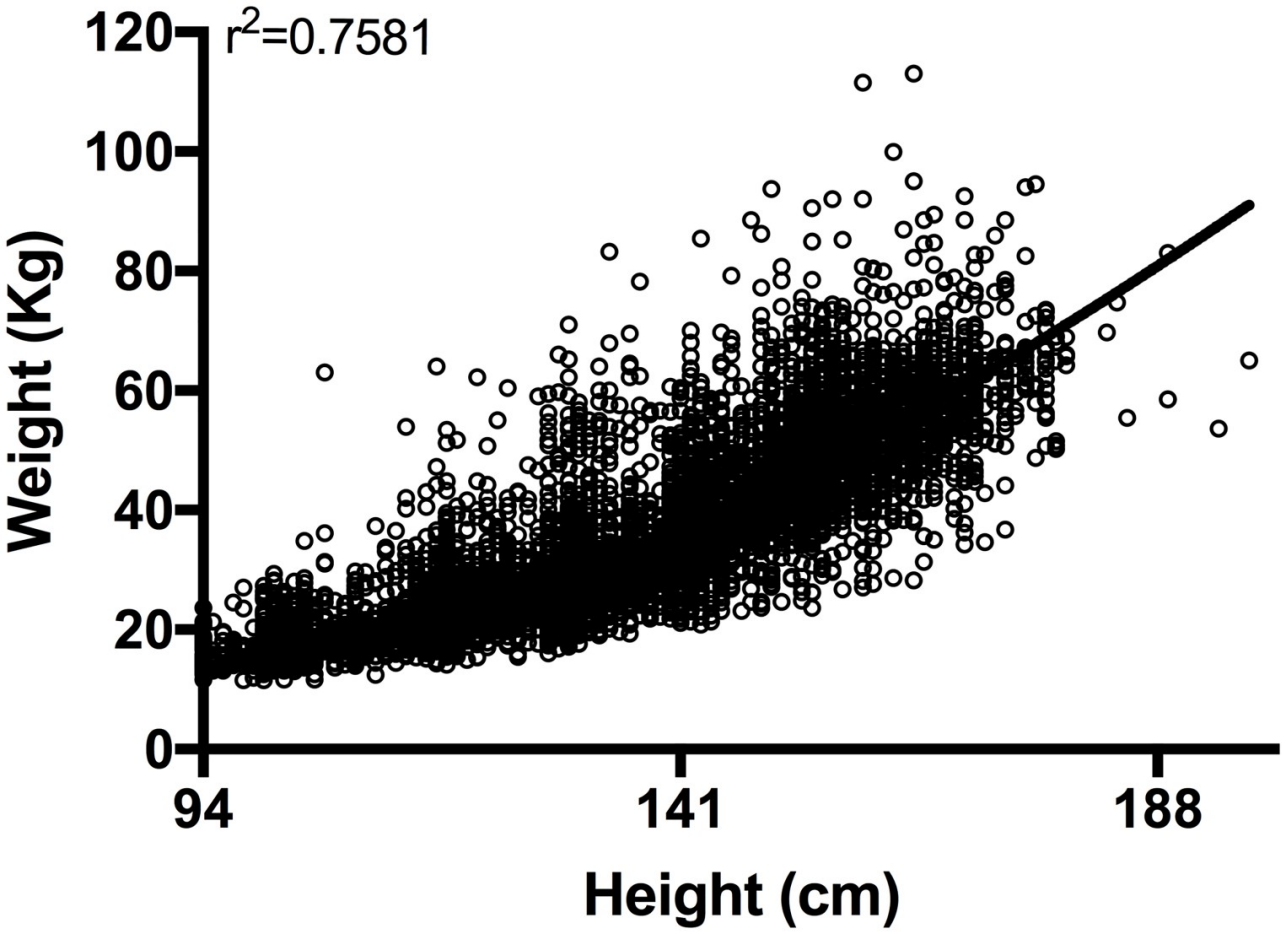
Distribution of height and weight measurements from the Mozambique study population (n = 9,827).

This research was conducted during the preventive chemotherapy phase of the SCORE study conducted among SAC and adults from 42 communities in five districts of Cabo Delgado province, Northern Mozambique (Fig 1) [16, 17]. Individuals were assessed in the school or at the household level during treatment whereby demographic and physical data (age, sex, height, and weight) were collected. All villagers were treated for schistosomiasis using the height-based dose pole. Weight was measured using a body digital scale (accuracy resolution 0.1Kg), calibrated according to the manufacturer’s recommendation (Tian Shan, China). In total, 10,611 individuals were surveyed. The WHO AnthroPlus software (WHO) was used to flag erroneous height/weight readings. 9,827 individuals’ data for height and weight, including SAC and adults (Fig 2), were included for analysis. Statistical analysis was performed using the statistical language R (version 3.3) (Lucent Technologies, USA). The package ‘tidyverse’ was used for data cleaning and summary statistics and ‘lme4’ for specifying generalized linear mixed models of the data. For the statistical comparison among the groups, we assessed the likelihood of underdosing by creating a binary variable, which was 0 for appropriate dose (as given by use of the WHO dose pole) and 1 for insufficient dose. This was specified as the dependent variable in a binomial generalized linear mixed model (GLMM), with ‘logit’ link function. Sex (m/f) and age group (5 to 8, 9 to 12, 13 to 15, 16 to 20, 21 to 55 and 56 to 95) were included in the model as fixed effects, and village and district were included as random effects to account for geographic clustering effects.

### WHO-dose pole vs. the modified-dose pole

In 2014, Palha de Souza, *et al*. [14] developed a modified dose pole by adding two height/dose intervals to correct for potential underdosing in adults. The new height cuts-offs of 156 cm and 164 cm allow for dosing of 3.5 tablets and 4.5 tablets, respectively.

For our secondary analysis, the relative accuracy of the standard and modified dose poles was calculated in terms of their capacity to provide a recommended dose between 40-60mg/Kg, considered to be optimal; 30-40mg/Kg, considered to be acceptable; < 30mg/Kg, considered to be insufficient; and finally, > 60mg/Kg, considered to be excessive. An appropriate dose was considered to be between 30-60mg/Kg. Moreover, the modified-dose pole results were also analyzed in combination with a body mass index (BMI) adjustment. For this analysis, adults with BMI > 25 kg/m^2^ were estimated to require an additional 25% of the average adult dose (2,400 mg), translating to one extra 600 mg tablet, as suggested by the modified-dose pole developers.

## Results

Of the total 9,827 individuals surveyed in northern Mozambique, 7,596 were SAC from 5 to 15 years old (4,141 male and 3,545 female), and 2,231 were adults between 16 to 95 years old (1,428 male and 803 female). As presented in Table 1, among 7,596 SAC, a total of 54.7% received an optimal dose (40-60mg/Kg) of praziquantel using the WHO dose pole, and 37.1% received a lower, but acceptable dose (30-40mg/Kg). Thus, 91.8% of the students in school surveys received what is considered an appropriate dose (30-60mg/Kg). 6.2% of the students received an insufficient praziquantel dose (< 30mg/Kg) whereas 2% received an excessive dose (> 60mg/Kg). Among the 7,596 SAC, the average and median doses were 41.4 mg/Kg and 41.2 mg/Kg respectively, and the minimum and maximum doses administered by the dose pole were 16.9 mg/Kg and 77.9 mg/Kg, respectively (Table 1).

**Table 1:** Demography of 9,827 individuals surveyed in northern Mozambique, by gender and age group, who received treatment with praziquantel doses below, within, or above the recommended amounts using the current WHO dose pole.

Age groups	Sample Size			Insufficient dose <30mg/Kg			Acceptable dose 30-40mg/Kg			Optimal dose 40-60mg/Kg			Excessive dose >60mg/Kg		
	T^a^	m^a^	f^a^	T (%)	m(%)	f(%)	T (%)	m (%)	f (%)	T (%)	m (%)	f (%)	T (%)	m (%)	f (%)
**School-aged children**															
**5–8 years old**	3040	1589	1451	7.4	7.3	7.5	44.3	46.4	42.0	46.3	44.7	48.0	2.0	1.5	2.5
**9–12 years old**	3382	1860	1522	5.0	4.9	5.0	30.2	31.0	29.3	62.3	61.8	62.9	2.5	2.3	2.8
**13–15 years old**	1174	692	482	5.4	4.6	6.4	38.1	36.7	40.0	54.5	56.8	51.2	2.0	1.9	2.3
**Subtotal**	**7596**	**4141**	**3455**	**6.0**	**5.8**	**6.3**	**37.1**	**37.9**	**36.2**	**54.7**	**54.4**	**55.1**	**2.2**	**1.9**	**2.6**
**Adults**															
**16–20 years old**	495	346	149	8.1	7.8	8.7	53.9	51.2	60.4	37.6	40.5	30.9	0.4	0.6	0.0
**21–55 years old**	1543	938	605	15.6	11.8	21.3	52.2	48.8	57.5	32.0	39.2	20.8	0.2	0.1	0.3
**56–95 years old**	193	144	49	10.9	11.1	10.2	52.3	50.0	59.2	36.8	38.9	30.6	0.0	0.0	0.0
**Subtotal**	**2231**	**1428**	**803**	**13.5**	**10.8**	**18.3**	**52.6**	**49.5**	**58.2**	**33.7**	**39.5**	**23.3**	**0.2**	**0.2**	**0.2**
**Community-wide (SAC and Adults)**															
**Total**	**9827**	**5569**	**4258**	**7.7**	**7.1**	**8.5**	**40.0**	**40.3**	**39.5**	**50.6**	**51.2**	**49.8**	**1.8**	**1.5**	**2.2**

^**a**^T = total; m = male; f = female.

**Table 2:** Modeling of the likelihood of underdosing based on binomial generalized linear mixed model (GLMM), considering gender and age groups as variables.

	Parameter	Adjusted Odds Ratio	P Value
**(Intercept)**	-2.97 (0.17)	0.05 (0.04, 0.07)	0
**Male**	-0.22 (0.078)	0.8 (0.69, 0.94)	0.0051
**5–8 years old**	0.36 (0.15)	1.43 (1.07, 1.93)	0.0167
**9–12 years old**	-0.03 (0.15)	0.97 (0.72, 1.31)	0.8508
**16–20 years old**	0.59 (0.21)	1.81 (1.19, 2.75)	0.0053
**21–55 years old**	1.32 (0.15)	3.73 (2.76, 5.04)	0
**56–95 years old**	1.06 (0.27)	2.88 (1.69, 4.93)	0.0001

When the performance of the dose pole was evaluated among 2,231 adults, it was found that 13.5% received an insufficient dose. When the percentage of insufficient dosing was disaggregated by gender, the frequency of adult females who were underdosed was 18.3%, in contrast to 10.8% for adult males (p = 0.005, Table 2). Ultimately, the most substantial difference by gender was found among adults aged 21 to 55 years (p < 0.001, Table 2), wherein 21.3% of females and 15% of males were underdosed. Among the adults, only 30% received an optimal dose (40–60 mg/Kg) of PZQ using the WHO dose pole, 51% received an acceptable dose (30–40 mg/Kg), and 5% an excessive dose (> 60mg/Kg). Among adults, the average and median doses were 37.3 mg/Kg and 37.0 mg/Kg, respectively, and the minimum and maximum doses administered by the dose pole were 15.3 mg/Kg and 63.1 mg/Kg, respectively.

When frequency of overweight/obesity (Body Mass Index (BMI) > 25.0) was evaluated among the Mozambican adults, the overall analysis revealed that 333 out of 2,231 adults (14.9%) could be classified as overweighed/obese. The peak rates of overweight among adults were among females aged between 21 and 55 years (23.5%) (Table 3).

**Table 3:** The percentage of adults surveyed in northern Mozambique, by gender and age, who were classified as overweight/obese based on body mass index values > 25.0.

Age groups	Sample Size			BMI >25.0		
	T^a^	m^a^	f^a^	T (%)	m (%)	f (%)
**16–20 years old**	495	346	149	43 (8.7)	33 (9.5)	10 (6.7)
**21–55 years old**	1543	938	605	261 (16.9)	119 (12.7)	142 (23.5)
**56–95 years old**	193	144	49	29 (15.0)	25 (17.3)	4 (8.2)
**Total**	**2231**	**1428**	**803**	**333 (14.9)**	**177 (12.4)**	**156 (19.4)**

^**a**^T = total; m = male; f = female.

Next, the performance of the WHO-dose pole was compared to that of the modified dose pole proposed by Palha de Souza and collaborators [14], using our database of Mozambican adult anthropometrics (Table 4). This modified dose pole was specifically developed to address the potential for underdosing in adults posed by the WHO dose pole. To re-study this problem, we used the height and weight data collected to determine the dosing prescribed by a modified dose pole and assess whether this would provide appropriate or insufficient doses (> 30g/kg or < 30g/kg). We created a binary variable for underdosing (0 for appropriate dose, 1 for underdose/insufficient), the dataset was filtered to consider only adults aged 16 and older, and the data were stacked so that there were two observations for each individual: One for insufficient dose (0 = appropriate /1 = insufficient) with the WHO dose pole, and one for insufficient dose (0 = appropriate /1 = insufficient) with the modified dose pole. We specified a GLMM with insufficient dose as the dependent variable. Fixed effects were sex, age group, and type of dose pole (WHO or modified). Individual ID was included as a random effect (variances from village and district level were accounted for by the individual ID random effect). The model indicated that the WHO dose pole was significantly more likely to result in underdosing for adult’s treatment than the modified dose pole (Table 5).

**Table 4:** The accuracy of the WHO standard dose pole compared to the modified-format dose pole, with or without BMI adjustment, in determining praziquantel doses for the 2,231 adults surveyed in northern Mozambique.

Age group	WHO-dose pole		Modified dose pole	
Adults (16–95 years old) n = 2,231	-BMI	+BMI	-BMI	+BMI
**Insufficient dose (<30mg/Kg)**	301 (13.5%)	274 (12.2%)	233 (10.4%)	219 (9.8%)
**Acceptable dose (30-40mg/Kg)**	1174 (52.6%)	1116 (50.1%)	983 (44.1%)	939 (42.1%)
**Optimal dose (40-60mg/Kg)**	751 (33.7%)	798 (35.8%)	1001 (44.9%)	1011 (45.3%)
**Excessive dose (>60mg/Kg)**	5 (0.20%)	43 (1.9%)	14 (0.60%)	62 (2.78%)
**Appropriate dose (30-60mg/Kg)**	1925 (86.2%)	1914 (86.9%)	1984 (88.9%)	1950 (87.4%)

The performance of the modified dose pole in reducing the underdosing of Mozambican adults was to decrease its frequency from 13.5% to 10.4%. In addition, it markedly increased the percentage of optimal dosing (40-60mg/Kg) from 33.7% to 45.3%. On the other hand, the modified dose pole combined with a recommended BMI correction adjustment did not provide any improvement in in reducing the number of potential underdose treatments (10.4% vs 9.8%).

**Table 5:** GLMM analysis to compare the odds of insufficient dosing (< 30mg/Kg of praziquantel) using the WHO dose pole vs the modified dose pole for adults in Mozambique.

	Parameter	Adjusted Odds Ratio	P Values
**(Intercept)**	-2.97 (0.17)	0.05 (0.04, 0.07)	0.000
**Masculine**	-0.22 (0.078)	0.8 (0.69, 0.94)	0.0051
**WHO dose pole**	3.72 (0.66)	41.12 (11.35, 148.96)	0.000
**21–55 years old**	0.62 (0.8)	1.85 (0.39, 8.89)	0.4403
**56–95 years old**	0.24 (1.3)	1.27 (0.1, 16.44)	0.8532

## Discussion

Schistosomiasis control is based on large-scale, repeated, and preventive chemotherapy treatment with PZQ, which has resulted in a significant reduction in its prevalence and associated morbidity worldwide. The WHO PZQ dose pole represents one of the key advances in the global initiative for schistosomiasis control. However, although it has a satisfactory accuracy for SAC treatment, it was not necessarily designed for accurate dosing of adults.

Currently, in some countries of sub-Saharan Africa, there are still reports of high endemicity areas for schistosomiasis (>50% of prevalence), requiring treatment of high-risk adults. [16]. The accuracy of the standard dose pole has been extensively tested for SAC treatment, showing very satisfactory effectiveness in delivering correct doses for 95% of SAC treated [10, 12]. Although it has satisfactory performance for SAC, there are discrepancies in efficiency in its current format, with much reduced performance in accuracy amongst adults [13, 14]. This is problematic, because adults are now identified as an important target for the control and elimination of schistosomiasis in highly endemic areas because of their contribution in the maintenance of active transmission within communities [23]. Moreover, although not assessed in this study, Recently studies have suggested a high risk of *Schistosoma* infections in early life [18], suggesting the need for inclusion of children ≤6 years in MDA programs. Coulibaly et al. [19] have reported the efficacy and safety of different dosages of praziquantel in preschool children, whereby there was a 72% cure rate with the 40 mg/kg dose and therefore endorsed preventive chemotherapy programs in children younger than 5 years of age. To include infants and PSAC in the MDA programs, an extended tablet pole was proposed, with two more height intervals: 60-83cm for ½ tablet and 83-99cm for ¾ tablet, and an upward revision of the 94cm threshold to 99cm [20, 21, 22]. This adaptation should lead to a new accuracy of 95.4% for all children (including infants), yielding an appropriate dose (30-60mg/Kg), with only 1.6% and 3% receiving an insufficient, or an excessive dose respectively [22].

Our study’s data collected in northern Mozambique supports the findings of other studies performed in South Africa and Zimbabwe [13, 14] that have suggested a need to implement an alternative way to deliver PZQ to adults on a mass scale. In 2007, WHO performed field-testing PZQ dosage for the treatment of opisthorchiasis in Lao PDR, comparing the accuracy of the dose pole with bathroom scales [24]. Using the Lao population’s height-for-weight data, and extrapolating it for the treatment of schistosomiasis, the study revealed that 18.7% of the adults (aged >15 years) would have received an insufficient dose of 30mg/Kg of PZQ using the dose pole. This is in line with the 10–20% dose pole under dosage for schistosomiasis that we and other have found among adults. In Mozambique, approximately 21% of adult females from 21 to 55 years old were found to have been underdosed. In light of the fact that several countries have conducted multiple rounds of MDA and are now moving towards potential elimination of schistosomiasis, this underdosing of an important subgroup of people may represent a significant risk for persistence of schistosomiasis. Considering their daily occupational exposures, such as collecting water, bathing, and washing in open water bodies, adults significantly contribute to the transmission potential in a community [17]. The results from the SCORE project in 150 villages in the province of Cabo Delgado in Mozambique in 2011, demonstrated that 44.8% of the surveyed adults were infected with *S*. *haematobium*, and out of the 44.8% infected, 7.1% were heavily infected, expelling ≥ 50 eggs per 10mL of urine [16]. This group of individuals must not be neglected by MDA programs because they may represent a key factor in maintaining transmission, while they are also in danger of developing advanced forms of *Schistosoma*-associated morbidity. In the Mozambique study villages, among 4,154 adults randomly surveyed in a knowledge, attitudes, and practices (KAP) survey, 3,971 (95.6%) stated that farming was their main occupation, washing (91.3%) and bathing (86.7%) in open water source were common practices, as was open defecation (83.3%) [17].

A common aspect in the dose pole studies in South Africa, Zimbabwe, Lao PDR, and Mozambique was the percentage of overweight and obese adults (BMI > 25), which occurred among 40.9%, 19.4%, 19% and 14.9% of adults, respectively. Adult females surveyed in Mozambique with an age range between 21 and 55 years demonstrated a peak of overweight/obesity of 23.5%. As reviewed by Ng and collaborators [25], the global prevalence of obesity has increased, affecting some countries more than others. According to the WHO [26], in 2016, more than 1.9 billion adults, 18 years and older, were overweight. Of these, over 650 million were obese. Thus, in affected countries, the accuracy of the height-based WHO dose pole for weight-adjusted schistosomiasis treatment might be dramatically reduced, because it functions under the presumption that a patient’s height and weight are closely correlated.

The resolution for this issue suggested by Palha de Souza et al. [14], and later supported by Baan et al. [13], was to modify the current dose pole by adding two height/dose intervals. Their modified dose pole was aimed at reducing underdosing in adults. Moreover, the authors also suggested a simple adjustment in the PZQ dose for individuals with overweight or obesity. These individuals, who could be classified by the community drug distributors using a pictogram [27], would require an additional 25% of the average adult PZQ dose (2,400mg), translating to one extra 600mg tablet. The use of the modified dose pole would have reduced the proportion of adult Zimbabweans receiving insufficient PZQ doses (< 30 mg/Kg), from 15% to 8.7%, and ultimately to 1.4% when combined with the BMI correction adjustment. Among South-African female students, underdosing would be reduced from 27% to 20%, and finally to 3.4% by adding the BMI correction. In the present study, use of the modified dose pole would have reduced underdosing in Mozambican adults from 13.5% to 10.4%, with a significant reduction from 21.3% to 15.5% among adult females between 21 and 55 years old. The reduction by the modified dose associated with BMI correction adjustment was not as great as in South Africa and in Zimbabwe, which could be explained by a lower rate of obesity among adults in Mozambique. Whereas concurrent food intake is known to affect PZQ uptake and ultimate blood levels [28], the impact of patient obesity (or pregnancy) on drug uptake and peak blood levels remains unknown. This will be a useful area for research in the near future.

**Fig 3:**
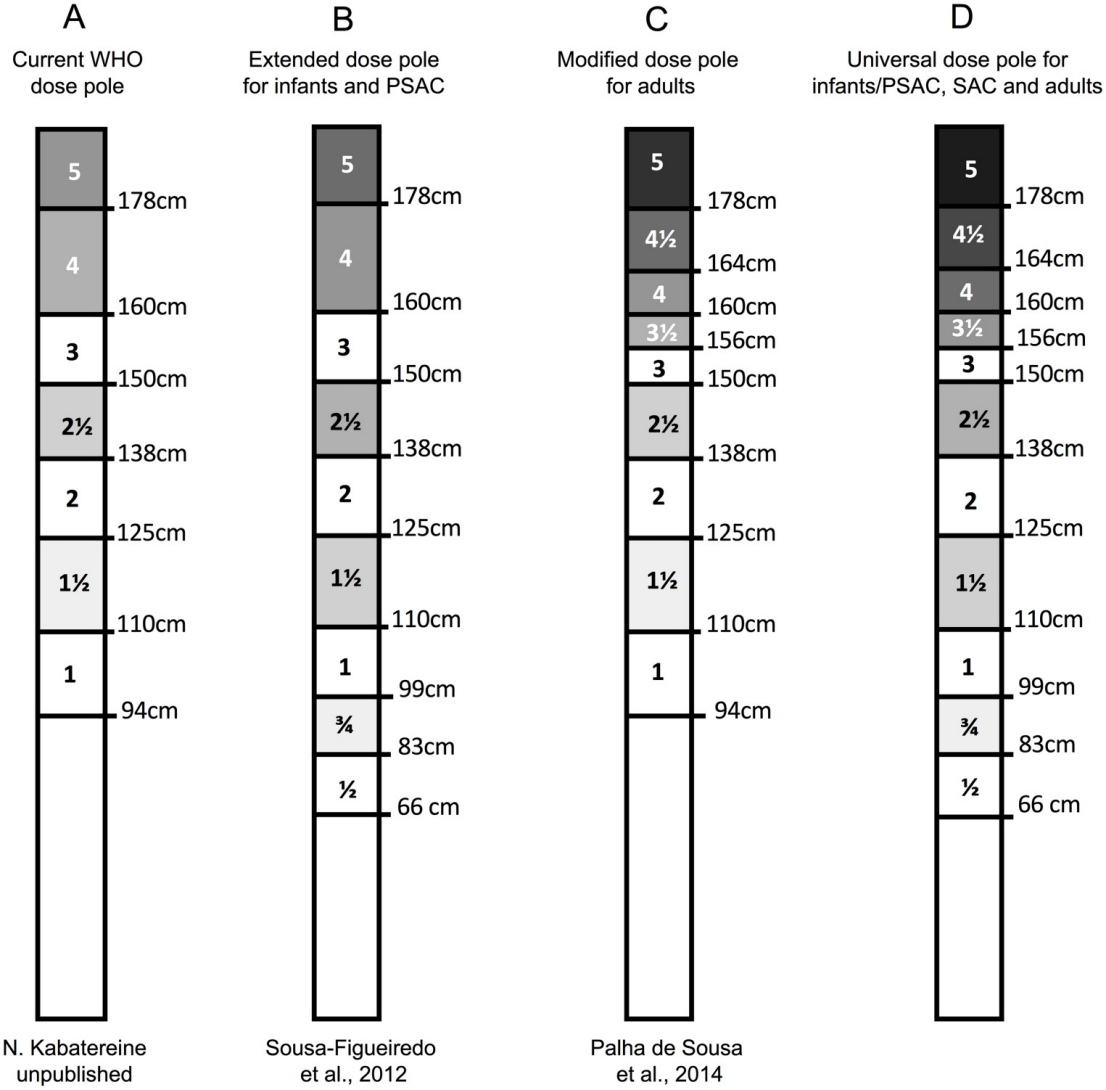
Current WHO-dose pole format (A), the extended dose pole for infants and PSAC proposed by Sousa-Figueiredo et al. [22] (B), the modified dose pole for adult treatment proposed by Palha de Sousa et al. [14] (C), and a universal dose pole for schistosomiasis treatment at all age groups (D).

In summary, particularly in the current climate where countries are moving towards elimination and community-wide preventive chemotherapy against schistosomiasis, we recommend a validation, based on an assessment of PZQ plasma concentrations, of the dosing recommended by the WHO standard dose pole. If significant underdosing is detected, to avoid large-scale administration of insufficient PZQ and its potential for emergence of drug-resistant strains, the WHO dose pole should be modified considering the implementation of a universal dose pole for all age groups to deliver praziquantel in endemic areas for schistosomiasis (Fig 3). Whilst we make this recommendations for a modified dose pole, we are similarly cognizant of the increasing burden for PZQ production that expansion to community-wide treatment programs is putting on pharmaceutical companies and donors alike. This should not however, take away from the need for accuracy particularly in the move towards elimination.
